# Decision-making for delivery location and quality of care among slum-dwellers: a qualitative study in Uttar Pradesh, India

**DOI:** 10.1186/s12884-016-0942-8

**Published:** 2016-07-07

**Authors:** May Sudhinaraset, Naomi Beyeler, Sandhya Barge, Nadia Diamond-Smith

**Affiliations:** Department of Epidemiology and Biostatistics, UCSF, 550 16th Street, Box 1224, San Francisco, CA 94158 USA; Global Health Sciences, UCSF, 550 16th Street, Box 1224, San Francisco, CA 94158 USA; Center for Operations Research and Training, 402, Woodland Apartment, Race Course Circle, Vadodara, 390 007 Gujarat India

**Keywords:** Maternal health, Child health, Urban health, Urbanization, India, Quality of care, Social support, Health providers

## Abstract

**Background:**

In 2013, the Government of India launched the National Urban Health Mission (NUHM) in order to better address the health needs of urban populations, including the nearly 100 million living in slums. Maternal and neonatal health indicators remain poor in India. The objective of this study is to highlight the experiences of women, their husbands, and mothers-in-law related to maternal health services and delivery experiences.

**Methods:**

In total, we conducted 80 in-depth interviews, including 40 with recent mothers, 20 with their husbands, and 20 with their mothers-in-law. Purposeful sampling was conducted in order to obtain differences across delivery experiences (facility vs. home), followed by their family members.

**Results:**

Major factors that influence decision-making about where to seek care included household dynamics and joint-decision-making with families, financial barriers, and perceived quality of care. Women perceived that private facilities were higher quality compared to public facilities, but also more expensive. Disrespectful care, bribes in the facility, and payment challenges were common in this population.

**Conclusions:**

A number of programmatic and policy recommendations are highlighted from this study. Future endeavors should include a greater focus on health education and public programs, including educating women on how to access programs, who is eligible, and how to obtain public funds. Families need to be educated on their rights and expectations in facilities. Future programs should consider the role of husbands and mothers-in-law in reproductive decision-making and support during deliveries. Triangulating information from multiple sources is important for future research efforts.

## Background

Globally, delivering in a facility has increased across Africa and Asia, among rural and urban, and rich and poor populations [1]. In India, facility deliveries have impressively increased from about 20 % to over 70 % in a span of 10 years; however this has not resulted in the maternal and neonatal health improvements expected by health policy analysts [2]. In 2013, the maternal mortality ratio in India was approximately 190 deaths per 100,000 live births and the infant mortality rate was 41 deaths per 1000 live births, one of the highest in the region [3]. Moreover, there is significant heterogeneity across states in India, with Uttar Pradesh, located in Northern India [4], reporting among the worst health outcomes in the country [5] and low levels of women’s autonomy [6].

National programs in India, such as the Janani Suraksha Yojana (JSY), have been the major drivers of increases in facility deliveries, particularly among the poorest populations. JSY, launched in 2005, is a conditional cash transfer program that financially incentivizes women to deliver in a facility. Women are offered approximately $15–$30, depending on the state in India. While facility deliveries have shown dramatic improvements, quality of care and challenges with the scheme continue, including ensuring skilled attendant at birth, experiences of disrespectful care in facilities and discrimination due to social status of women, transportation barriers to facilities, and low levels of trust in public health facilities [2]. Historically, much of the maternal health efforts have focused in rural areas; however, in 2013, in response to growing urban health disparities, India launched the National Urban Health Mission (NUHM), which focuses on meeting the healthcare needs of the urban poor, particularly slum populations.

Nearly 100 million people in India live in slums: 17.4 % of all urban households, reaching as high as 41.3 % of the population of Mumbai. Barriers to healthcare utilization for maternal health in slum communities are well documented in the literature. Studies find that while slum dwellers prefer formal over informal maternal health services, there are significant barriers to accessing care, including geographical access, ineffective family household decision-making, safety concerns, high cost of health services, and perceived low quality of providers [7]. Existing literature in Africa and parts of Asia suggests that slum-dwellers experience a number of adverse maternal and child health outcomes, including less access to antenatal care (ANC) services and facility deliveries. Urban populations have grown rapidly over the past two decades; it is estimated that by 2025 64 % of people in low-income countries will also live in urban areas [8]. This process of urbanization presents a number of new health opportunities and challenges: although urban areas can provide residents with greater access to health and economic resources, urban residents are also exposed to new economic and environmental risks. This is particularly true in slum areas.

Given the renewed focus on urban slum populations, it is important to better understand the healthcare challenges that this population faces, including decisions around where to deliver, support structures, financial challenges, and perceived quality of care. Few studies have gathered voices of women, husbands, and mothers-in-law in order to triangulate information [9]. Past qualitative studies have found that financial burdens restrict place of delivery to public hospitals, and that cultural and social factors, such as expectations around respectful and safe care, were significant drivers for location of delivery. More recent studies also find that mothers-in-law, particularly paternal mothers-in-law, play a significant role in household decision-making, including health services [10]. The role of husbands and men in particular has been under researched, but studies find that interventions targeting men during the time of delivery have positive health benefits to the mother and child [11]. There is little information on decision-making among women, and how families, including husbands and paternal and maternal grandparents, navigate the continuum of maternal health services from antenatal care (ANC) to a facility delivery in slum areas. A recent qualitative study in rural India attempted to include information from recent mothers, their husbands and their mothers-in-laws and found that family members play an important role in where women seek delivery care [12]. This study, however, was not able to link family members and therefore limited in understanding how household dynamics may influence shared decision-making. Triangulating these different voices and perspectives may shed light on points of interventions to improve maternal and child health in India, particularly in urban settings given rapid changing gender and social norms.

Through qualitative interviews, this study seeks to examine the ways in which household decision-making influences the health utilization of maternal health services in urban slums and offers insights for points of interventions for the health sector. This study aims to understand the principal economic, environmental, and social factors that influence women’s decisions to seek maternal health services and the quality of care received. This study first addresses decision-making around where to deliver, including home vs. facility or public vs. private sector; it then delves into recent mothers’, husbands’, and mothers-in-laws’ experiences with maternal health services.

## Methods

### Study participants and procedures

In total, we conducted 80 in-depth interviews, including 40 with recent mothers, 20 with their husbands, and 20 with their mothers-in-law. Purposeful sampling was conducted in order to obtain differences across delivery experiences (facility vs. home), followed by their family members. We recruited 30 women who delivered in a facility and ten women who delivered at home. Two recruitment lists were formed to provide the sampling frame for interviews. First, researchers worked with Anganwadi centers in the two study sites to develop lists of women who delivered in the past year. Second, researchers went door-to-door in the communities to recruit more women who had delivered in the past year in order to ensure that a variety of women were recruited from the community. Study participants were recruited from two slums in Uttar Pradesh, Lucknow. Inclusion criteria for recent mothers included women who delivered a child in the past year. Once women were recruited, they were asked whether their husband or mother-in-law lived in the same house and available to speak, and only if the woman gave permission did we recruit their partners and mothers-in-law.

Interviews lasted approximately one hour, were tape-recorded, transcribed into Hindi, and translated into English. Four trained research assistants, including two men and two women, facilitated the interviews. Interviewers and participants were matched according to gender. All interviews were conducted between April and July 2014. Participants were asked a number of questions including about their delivery experiences, quality of care received, choice of provider and delivery location, support received before, during, and after the time of delivery, cost of care, and decision-making about maternal and child health services. Women, husbands, and mothers-in-law were asked similar questions in order to triangulate information where possible.

### Data analysis

Data was analyzed using Atlas-ti software by two trained researchers. Content analysis was used to analyze the textual information. The analysis was completed in three phases. First, two researchers read each transcript several times to examine the text as a whole, and to identify initial impressions. Second, two researchers then coded a subsample of the text in an iterative process. Two researchers first developed codes separately using ten transcripts, revised and refined codes together, and developed a common coding scheme. The tentative codes were discussed and revised by the researchers. The codes were then applied to all of the transcripts. Finally, codes were grouped into families, or broader themes. Researchers had discussions regarding broader themes, including where participant voices converged and diverged. Matrices were developed for specific themes in which a variety of participant types were questioned.

### Ethical approval

The study and all accompanying study materials were reviewed and approved by the Institutional Review Boards at the University of California, San Francisco (UCSF) and the Centers for Operations Research and Training (CORT) in Gujarat, India. Verbal informed consent was obtained from all study participants.

## Results

### Participant demographic characteristics

The women interviewed for this study were, on average, 26 years old (Table 1). In terms of religion, the large majority (*N* = 37, 93 %) of respondents were Hindu, and the rest were Muslim. Fifty percent (*N* = 20) belonged to a Scheduled Caste, 20 % (*N* = 8) to Other Backwards Caste (OBC), and 30 % (*N* = 12) to none of these. All women were married and currently living with their husbands, and 65 % also had their mothers-in-law living with them (*N* = 26). Twenty-eight percent had no education (*N* = 11), 23 % had less than 8 years (*N* = 9), 38 % had 8 to 11 years (*N* = 15) and 10 % had 12 or more years of education (*N* = 4). Women had, on average, 1.9 births in their lives, ranging from 1 to 7. Women spent an average of 7,721 rupees on services, supplies and medicines during their most recent delivery (interquartile range from 0 to 12500), and a mean of 334 rupees on transportation to the facility (IQR 0-200). The majority (*N* = 35, 88 %) were not currently working outside the home. None of the women had been living in Lucknow their entire lives, 8 % had lived there less than 1 year (*N* = 3), 20 % for 1 year (*N* = 8), 40 % for 2–5 years (*N* = 16), 18 % for 5 to 10 years (*N* = 7) and 15 % for more than 10 years (*N* = 6). The majority (*N* = 32, 80 %) had lived in a city before moving to Lucknow, 5 % in a town (*N* = 2) and 15 % in the countryside (*N* = 6). The majority of women had their most recent birth at a large government hospital (48 %), followed by 25 % at home (*N* = 10), 25 % at a private hospital (*N* = 10), and 2 % at a lower level facility (*N* = 1).

**Table 1:** Demographic Characteristics of Study Participants

	Women (*N* = 40)	Husbands (*N* = 20)	Mothers-in-law (*N* = 20)
Age, mean (IQR)	26 (23–29)	30 (25 to 32)	54 (45–61)
Hindu (N (%))	37 (93 %)	19 (95 %)	18 (90 %)
Caste			
Scheduled Caste	20 (50 %)		6 (30 %)
Scheduled Tribe	0		0
Other backwards	8 (20 %)		7 (35 %)
None	12 (30 %)		7 (35 %)
Education			
No education	11 (28 %)	2 (10 %)	16 (80 %)
Less than 8 years	9 (23 %)	9 (45 %)	3 (15 %)
8 to 11 years	15 (38 %)	9 (45 %)	1 (5 %)
12 or more years of education	4 (10 %)	0	0
Occupation			
Not working outside the home	35 (88 %)	1 (5 %)	n/a
Unskilled/skilled	2 (5 %)	9 (45 %)	
Services	2 (5 %)	5 (25 %)	
Sales	0	5 (25 %)	
Professional, technical, or managerial	1 (3 %)		
Migration Status			
Entire lives	0	13 (65 %)	3 (15 %)
< 1 year	3 (8 %)	0	0
1 year	8 (20 %)	2 (10 %)	0
2–5 years	16 (40 %)	0	1 (5 %)
5 to 10 years	7 (18 %)	1 (5 %)	2 (10 %)
More than 10 years	6 (15 %)	4 (20 %)	14 (70 %)
Place of residence before migration			
City	32 (80 %)	7 (100 %)	15 (88 %)
Town	2 (5 %)	0	0
Countryside	6 (15 %)	0	2 (12 %)
Number of living children, mean (range)	1.9 (1–7)	n/a	n/a
Place of most recent delivery, N (%)		n/a	n/a
Government Hospital	19 (48 %)		
Private Hospital	10 (25 %)		
Lower level Facility	1 (2 %)		
Home	10 (25 %)		
Mean cost spent on delivery	7,721 (0–12500)	n/a	n/a
Mean cost spent on transportation to delivery	334 (0–200)	n/a	n/a
Widowed (Mother-in-laws only)	n/a	n/a	6 (30 %)

Husbands were, on average, 30 years old. The majority of husbands were Hindu (*N* = 19, 95 %). In terms of educational attainment, 10 % had no education (*N* = 2), 45 % had 1–7 years (*N* = 9), 45 % had 8 to 11 years (*N* = 9) and none had 12 or more years of education. Almost half (45 %) worked as unskilled or skilled laborers (*N* = 9), 25 % worked in services (*N* = 5), 25 % worked in sales (*N* = 5) and 5 % were not working (*N* = 1). Sixty-five percent of the husbands had been living in Lucknow their entire lives (*N* = 13), 10 % for 1 year (*N* = 2), 5 % for 5-10 years (*N* = 1) and 20 % for more than 10 years (*N* = 4).

Mothers-in-law were, on average, 54 years old. All but two (10 %) were Hindu while the others were Muslim. The majority (*N* = 16, 80 %) had no schooling, 15 % had 1–7 years (*N* = 3) and 1 (5 %) had 8–11 years. The majority (*N* = 14, 70 %) had been living in Lucknow for more than 10 years, 15 % had been living there their whole lives (*N* = 3), 1 (5 %) for 2–5 years and 10 % for 5–9 years (*N* = 2). Most of the mothers-in-law who had migrated came from a city (*N* = 15, 75 %) and 12 % from the countryside (*N* = 2).

#### Decision-making about delivery location

Throughout the course of pregnancy, women and their families made a number of decisions regarding where to seek health services for antenatal and delivery care. In making decisions about where to deliver, respondents made choices about delivering at home or in a facility, and for those respondents who delivered at a facility, made choices about delivering at a public or private facility. These decisions were influenced by multiple factors, including prior experience of the woman or her family in receiving maternal health care, perceived accessibility of the facility – based on both cost and proximity—and perceived quality of the facility. Intra-household dynamics and relationships between the woman, her husband and mother-in-law also influenced how these decisions were made.

### Household dynamics: joint decision-making common

Few of the women respondents made decisions about where to seek maternal health services on their own; rather these decisions were most commonly made together by the woman with her husband, mother-in-law, or both. These dynamics varied across households, with some families reporting that the mother-in-law was the primary decision-maker regarding maternal health care seeking, while in a smaller subset of families the woman and husband made these decisions independently of the mother-in-law. Among women who delivered at home, the majority reported that their mother-in-law was the primary decision-maker about where to deliver, while women who delivered in facilities were more likely to report that their husbands or they themselves were the primary decision-maker about where to deliver.

In most cases, the previous experiences of other family members, including sisters, sisters-in-law, mothers, and mothers-in-laws, at different health facilities were important factors in care-seeking decisions. For example, as one mother-in-law explained regarding her decision to send her daughter-in-law to a particular hospital:

*My sister-in-law’s children were also born there. My daughter’s children as well as my elder sister-in-law’s children were also born at [that] Hospital. My eldest grandson was also born there. (Mother-in-law, age 46)*

Similarly, a woman also explained her decision to attend a particular hospital based on her sister’s prior positive experience receiving maternal care there.

*My sister also went there for her delivery. From her pregnancy to delivery, she went there for all types of treatment. So on her suggestion, I went there. (Woman, age 34, 1 child)*

Choice of place of delivery was influenced by other people’s experiences as well as their own perceptions of convenience, cost and quality.

### Home or facility delivery: complications, costs, and perceived quality

The majority of respondents (75 %) had their most recent delivery at a hospital or facility. The most common reason mentioned for selecting a facility for delivery, rather than home, was that facilities were considered a safer location for care in case of an emergency. As one respondent explained, her mother-in-law decided that she should go to a facility for the following reasons:

*Yes, she [mother-in-law] had also said that it is better to be in hospital --what if you start feeling unwell suddenly, or something else, require some machine, something, then all these facilities are there, at home you wouldn’t find all this (Woman, age 27, 2 children).*

However, despite the perception that facilities offered a safer location for delivery, a significant number of respondents (25 %) delivered at home. Among families who chose to have a home delivery, women, husbands, and mothers-in-law discussed two main reasons for this decision. The first, and most common, reason for choosing home delivery were the financial barriers to facility delivery, including both the cost of delivery services as well as the indirect costs associated with seeking treatment outside of the home such as for transportation.

*If [delivery] is done somewhere else like hospital, it costs a lot of money. It can be done at home for less money. And it is not possible to give proper care at the hospital, which can be done at home… It was right to do it at home, because we do not have so much money to take her to the hospital. They require money everywhere. Who’s going to give? It costs over twenty thousand rupees, how can we do it, tell us? That is why we do it at home. If it costs twenty thousand, from where do we get that kind of money? (Mother-in-law, age 60)*

*There was a rumor in the village that I might have to pay after delivery—they [facility] might charge something. I am not able to pay; we don’t have that much money. So, I didn’t go there ever again….I am afraid that’s why I didn’t visit hospital; it’s good to bear pain and expenses in home; at least I’ll be happy at home. (Woman, age 35, 5 children)*

In addition to concerns about the cost of delivery services in facilities, many families reported that the indirect costs associated with seeking care in a facility were prohibitive to having a delivery outside of the home. The main concerns were regarding the difficulties and cost of arranging transportation to the facilities, and in arranging childcare for other children at home while the mother was at the facility.

*We are poor and I have small children. If I stayed at home I would be able to take care of children and other thing, but if I stayed at hospital, how would my mother-in-law manage all these things? (Woman, age 35, 5 children)*

The second main reason respondents reported choosing to deliver at home was a concern about the quality of services provided at facilities. For many women delivering at home, the main quality concerns were about the way they would be treated at the facility, specifically fear of being disrespected, ignored, or treated poorly. As mentioned above, the prior experiences of family members and neighbors played a significant role in shaping these perceptions and influencing these decisions.

*Someone in the neighborhood told her [my mother-in-law] that if we cry and howl too much in the hospitals, they start hitting us. She got scared that ‘if my daughter-in-law gets troubled and if it is painful she will cry, and at that time if any nurse hits her.’…So she said it will be good if I deliver at home. (Woman, age 27, 2 children)*

Many families who selected home delivery due to their concerns about how they would be treated at a facility indicated that these concerns were related to issues of class and social status.

*Rich people always get preference over poor. Poor people always stand in line while rich people get the direct entry. Poor people always suffer. That’s why many poor people hesitate to go to a facility…(Woman, age 35, 5 children)*

For families preferring home delivery, it was this association between poor treatment and their social status that led some families to avoid seeking care at hospitals, in combination with the concerns about the cost of delivery and associated costs of seeking treatment outside the home.

### Public or private facility: perceived higher quality in private sector

Similar to decisions about home or facility delivery, the perceived quality and cost of services were main determinants in selecting the type of provider—either public or private. In general, women, mothers-in-law, and husbands perceived private facilities to offer higher quality services than public facilities. This was based on the perception that public facilities had longer wait times, poor interpersonal treatment, and lack of essential infrastructure.

*Private is best....In government there are no services. We have to stand in the queue for two hours, then your turn comes.....they do a simple examination and then inform you to come again day after tomorrow…. If you go to private, only money will be spent. Facilities will be availed quickly. (Husband, age 28, 2 children)*

One husband described trying to go to a public facility when his wife was in labor, but after waiting for hours with no attention, taking her to a private facility, where she got attention quickly.

*When we went there [public hospital], they admitted my wife inside. We sat there for almost 2-3 hours, my wife was suffering…I said it is no point tell me a place where I can take her..no one paid any attention to us, then I picked up my wife and got her to [private hospital]..then at [private hospital]..the doctor said for operation. (Husband, age 23, 1 child)*

However, private facilities were generally considered more expensive than public facilities. For this reason, even among respondents who reported that private facilities offered higher quality care, many chose to deliver in a public facility because of the high cost of private care. For some, delivering in a public facility enabled them to save money that they could use to attend a private facility if there was an emergency that required higher-level care.

*Yes, they said “come on daughter let’s go there in government hospital, if we go to private, it will be more expensive.” And there was not much problem. If problem is there, then to save oneself a person can go to private. We go to private if there is any problem. (Woman, age 26, 2 children)*

When families could afford the cost of private care, or in cases when a woman was thought to require emergency or higher-level treatment, families made the decision to seek care in private facilities. However, in general, the overwhelming sentiment that private facilities offered higher quality services was not matched by a strong preference to seek care in these facilities given the cost barriers that were not present at public facilities.

#### Experiences in facilities

In addition to the challenges women and their families experienced in accessing care, those who delivered in a facility also expressed a number of concerns about the quality of care they received. These concerns, similarly to the factors that influenced women’s decisions about where to seek maternal health care, centered on quality and cost. The primary concern about quality was the poor treatment many women and family members reported; while the concerns about cost centered on the common practice of facilities requiring bribes or informal payments to secure treatment.

### Disrespectful care common

Women at both public and private facilities expressed dissatisfaction with the quality of interpersonal care at the facilities, in particular being treated disrespectfully by nurses and other health providers. Many women reported being yelled and shouted at; for example, one woman said there was “a lot of shouting and beating” (Woman, age 28, 1 child). The family members also reported disrespectful care from providers. Doctors told this same woman’s husband that he and his family could not be near the woman while she was in labor.

*The person who does delivery was speaking very rudely…they asked us to “get out from here, why are you gathering here, move, get out.” We were not allowed to stand outside even. (Husband, age 40, 1 child)*

These concerns about disrespectful treatment from providers were reported equally across both public and private facilities. Although some women did report that they felt they were treated better in private facilities, many respondents indicated that there was no difference between type of facility and that disrespectful care was common across all facilities.

*Wherever it is, in government hospital, all government hospitals and nowadays in private hospitals also, they say bad words....Doctor, no its nurse, she says inappropriate words and bad words. (Woman, age 30, 3 children)*

However, as indicated above in the discussion about home delivery, there was a broad feeling that disrespectful treatment at facilities could be largely attributed to issues of social and economic status. As one husband explained:

*Only if you have resources, or influence in the government, only then will you be listened to [at the facility]. Wherever you go-- private or government hospital-- you will be asked who are you, what do you do. If you say you work as a laborer, then no one will give you any attention. (Husband, age 24, 1 child)*

It is important to note that not all respondents had negative experiences during their facility stays, with some reporting their treatment and experience to be positive. Although in some cases, this was acknowledged with a level of surprise, or seen as the exception to standard treatment practices.

*Their services were very good. I mean whether it was staff related, doctor related. From conversation to everything was very good. My experience was very good. (Woman, age 34, 1 child)*

### Financial barriers: bribes and tokens paid to facility staff

Respondents reported a number of financial barriers faced while seeking maternal health services. The primary challenge reported was the common practice of providers and other people at the facility seeking bribes or payments above the formal fees prior to providing essential care and services. Many respondents perceived the poor treatment by providers as linked to this system of informal payments; according to one woman, *“You have to give them [facility providers] bribes in order for them to attend to you” (Woman, age 30, 3 children).* Several respondents indicated that bribes were common throughout the hospital experience, and others reported that they were denied care if they did not provide financial “tokens” to facility staff.

*The nurses demanded money.... I had a baby girl and after the delivery they kept her on the scale and demanded Rs.300-400. If I will not pay money they will not return my baby to me. We are poor and how will we have this much money to give? (Woman, age 26, 3 children)*

*The gate peon was asking for money, and when baby boy was born I was sitting outside the operation theater when I went to get him they were asking that we give money so we give Rs.600. We took the baby boy and went upstairs for cleaning. There, everyone was asking for money – nurse, maid, everyone. Then they shifted her to another room, there were two people on the same bed…they asked for money again to shift her to an empty bed, then again for taking her to the room. So we gave money at 6-7 places…everywhere they asked for money. (Mother-in-law, age 50)*

These payments, and the informal format in which they were requested without families being able to anticipate the cost of services, was a source of both financial and emotional stress for many respondents.

### Financial barriers: payment challenges and difficulties with JSY payment

Respondents highlighted two additional sources of financial stress associated with a facility delivery. The first was the need to borrow money to cover the cost of services. Many respondents, both women and husbands, reported selling gold from their dowry or other household goods, or borrowing money from family or community members, in order to pay for their hospital stays.

*How did we bear the expense? As my husband is the only earning member and there are 8 people dependent on him, so we had to sell some of our household goods. (Woman, age 35, 5 children)*

*That arrangement- I had taken advance from my employer and also borrowed some money from my relatives…My people are really nice, my relatives, my boss, they did not show any reluctance to give the money..[they] gave money with ease. (Husband, age 23, 1 child)*

While for some families, borrowing money was a feasible option, for others the need to borrow money was seen as a burden. This was particularly true for those families who borrowed with the expectation of repayment, when this repayment would mean a significant financial burden for the family.

*We had to borrow money…We did not feel nice. We had thought that in government hospital everything is free, so we won’t have to pay anything. But they referred us to private hospital, so we had to borrow money, which made us sad. (Woman, age 23, 1 child)*

The second challenge women reported was in receiving the JSY conditional cash transfers. All women participants in this study were eligible to receive JSY payments; however, only 12 of the respondents reported receiving JSY, and of these, 4 of them reported not having cashed it yet. The primary reason respondents gave for their non-participation was the requirement to open a bank account in order to enroll in the JSY program. The cost of opening a bank account was reported to be equivalent to the amount of the JSY payment, reducing respondents’ motivation and interest to participate.

*I will be able get it [bank account] opened with one thousand rupees. And we will get Rs. 1000 Rupees. So what is the benefit of getting it opened? (Woman, age 20, 1 child)*

Among women who did receive JSY payments, many reported challenges in receiving the funds, such as having to wait many hours to receive the check or having to pay large bribes to hospital staff in order to receive their payment.

*The service that we got, the cheque, we gave it all away. Everyone was asking for money, the nurse or the helper, everyone was asking for money. We gave them more than what we got. (Woman, age 20, 1 child)*

Many respondents also reported challenges when trying to cash the JSY payments, specifically due to not having the required identification for processing the payments.

*I received a check but it is still not cleared. For clearance they are asking for [identification card]. They had given 3 months time, but to date I have not received the pehchan card, and the check is still lying with me uncleared. (Woman, age 26, 3 children)*

*We had to get this scheme but we did not have any proof. They said if you have any residence proof or ID proof or anything, we did not have anything so we would have got the facility if we would have tried we would have got the money but we did not avail it, so we would say it is our mistake that we did not go again and try to get that money…When my first child was born I was given a voucher. We have to take it to the bank, I went there twice, [my husband] went twice to the bank and we spent 250 – 300 rupees as well but we did not get the money. Now I still have the cheque with me. (Woman, age 22, 2 children)*

The association respondents made between these payments and the quality of care they received, as well as their social and economic status, was an additional source of concern and stress.

## Discussion

This study is important because it triangulates voices from recent mothers, their husbands, and mothers-in-laws and sheds light on potential interventions for improving maternal health outcomes in slums in India. The purpose of this study was to identify women’s motivations for delivering in a facility compared to their homes and to identify their experiences with care. Findings from this study suggest that the negative experiences women and their social networks face at the time of delivery were mostly related to financial challenges and quality of care. In particular, disrespectful care deterred some women from delivering in facilities and influenced their decisions to deliver at home vs. the facility. Our findings support other recent qualitative work in Karnataka, India, that found that perceptions of quality related to disrespect and cleanliness, as well as the costs of delivery, were main factors determining place of delivery and keeping women out of facilities [9]. A recent systematic review explored multiple domains of mistreatment that women experience during childbirth and found that mistreatment is prevalent in many countries across the globe [13]. Women in our study reported being shouted at, verbally abused, and being discriminated against because of their status. Our findings support this body of literature on mistreatment and disrespectful care, and provide additional evidence that these experiences, both those personally experienced and the experiences of others, impact decision-making and keep women from seeking care in facilities with trained providers.

This study also highlighted the large burden on families from the costs of delivery, including bribes for providers and facility staff. Past research has highlighted that high out-of-pocket costs are one of the main barriers to women delivering in a facility or with a trained provider in India [14]. Although the JSY program should have been available to all of the women in our study, many did not receive the benefit, or if they did, were not able to actually cash it or ended up giving most of it away to pay for additional bribes or costs of the delivery. Other studies have highlighted how the JSY program is not able to cover all costs associated with delivery, and in fact might have led to more out-of-pocket expenditures [15]. Fears of costs were one of the primary deterrents from going to a facility, and also impacted decisions about private versus public facilities. Families resorted to taking loans or selling goods, leading to long-term impacts from the delivery on the family. The JSY program is structured to be an incentive to delivering in a facility, eliminating cost barriers associated with delivery services. It is clear that the JSY program must find ways to ensure that women and families actually are able to receive the benefit. Additionally, finding ways of eliminating the extra fees that families are being asked to pay is critical, including holding facilities accountable and punishing facilities that require extra payment.

Our findings also highlight the importance of other family members of the decision-making process about where a woman delivers. Most research exploring choice of delivery focus on women’s individual characteristics, or sometimes those of her husband; however, it is clear from our findings that these decisions are most often made at a family or couple level [16]. Past research has shown that women’s autonomy is an important factor on her health care utilization, and that women who live in nuclear households were more likely to deliver in a facility than women living in co-resident households (with in-laws) [17, 18]. Our qualitative data help explain these findings, since we found that women were more likely to deliver in a facility in households where women, or women and their husbands, were the main decision-makers.

There are a number of limitations to this study. First, as is common in qualitative studies, the sample size is small and therefore not generalizable to other areas outside of this study site. It is important to note that there is significant heterogeneity across slums in India including differences in poverty levels, size, and population, race, and migration mix. This study describes the specific experiences of individuals living in particular slums in Lucknow, Uttar Pradesh. The sampling frame for the study attempted to reach both women who went to anganwadi centers as well as women in communities in general, with the goal of highlighting different types of women and families. Second, while we attempted to sample a mix of women who delivered in a facility and at home, other experiences, including levels of complication may influence results and were not captured systematically in this data. For example, women who experienced complications at the time of delivery may report more negative experiences compared to women without complications. Finally, eligibility for the study includes women who had delivered in the past year. This may lead to recall bias, as 1 year may be a long time for women, their husbands and family members to recall their decision regarding delivery and experiences of care.

Despite these limitations, this study points to a number of programmatic and policy implications for urban slum populations. From a policy perspective, while women were aware of the JSY program, few women accessed this program. Therefore, targeted education in these communities regarding different public programs is warranted, including how to sign up for the program, how to receive incentives, and who is eligible. Follow up regarding how to access funds is particularly important. Future programs should include husbands and mothers-in-law in the education process. Many times, decision-making on where to deliver occurred in the context of families and social networks; therefore, programs should engage these broader contexts in health education and promotion. This may include partner communication counseling or educating mothers-in-law on the benefits of delivering in a facility.

Finally, women in the study suggested that disrespectful care occurred in all types of facilities in India, both public and private facilities. This represents a significant challenge in India. Because experiences of disrespectful care were common in this population, future programs should focus on patient-provider interactions among urban, slum women in particular who may face discrimination due to their lower status. The World Health Organization (WHO) has outlined strategies to address disrespectful care which includes support through a companion of choice, access to foods and fluids, ensuring confidentiality and informed choice, and assuring high quality information for women to make informed decisions about care [19]. Other strategies to work with low-status women may include working with facilities to improve women-centered quality of care, training providers on culturally competent care, and involving women and their families in care processes.

## Conclusions

Financial barriers and disrespectful care were the main challenges experienced in urban, slum populations in regards to maternal health services. A number of programmatic and policy recommendations are highlighted from this study. Future endeavors should include a greater focus on health education and the JSY program, including educating women on how to access the program, who is eligible, and how to obtain funds. Families need to be educated on their rights and expectations in facilities. Future programs should consider the role of husbands and mother-in-law in reproductive decision-making as well as improving respectful care in facilities

## Abbreviations

ANC, antenatal care; JSY, Janani Suraksha Yojana; UCSF, University of California, San Francisco
